# What and who? Mindfulness in the mental health setting

**DOI:** 10.1192/pb.bp.116.054122

**Published:** 2016-12

**Authors:** Tamara A. Russell, Gerson Siegmund

**Affiliations:** 1 Institute of Psychiatry, Psychology and Neuroscience, King's College London, UK; 2 Federal University of Rio Grande do Sul, Brazil

## Abstract

A strong and growing evidence base exists for the use of mindfulness-based interventions to prevent relapse in major depression and for the self-management of chronic physical health conditions (e.g. pain), but the evidence in other domains of mental health work is still emerging. Much work is being conducted outside the evidence base and standardised protocols, and by individuals with varied levels of experience and training. The (mis)perception of mindfulness as a ‘simple technique’ belies the complexity and skill needed to deliver a mindfulness training that has real therapeutic and transformative power. We propose a framework to help clinicians think through the suitability of mindfulness for their particular client group with the intention of providing guidance for thoughtful decision-making.

Much of the current research on the clinical efficacy of mindfulness-based interventions (MBIs) is based on two standardised programmes: mindfulness-based stress reduction (MBSR) and mindfulness-based cognitive therapy (MBCT). These facilitated, closed, 8-week group training programmes are delivered to those with chronic physical health conditions (in the case of MBSR) and recurrent depression (MBCT). Some studies have evaluated adaptations of these protocols for other clinical populations,^1,2^ and a handful have tested the feasibility of novel protocols.^3,4^ The existing meta-analyses for both MBSR and MBCT^5-12^ report medium to large effect sizes across a range of outcome measures (including clinical ratings, quality of life, mindfulness and cognitive change), with good evidence that these MBIs can reduce anxiety and depression.^8,9,13^ A UK Mindfulness All-Party Parliamentary Group review^14^ has recently recommended that MBCT be available within the National Health Service (NHS) for adults at risk of recurrent depression and that it should be accessible to everyone with a long-term physical health condition and recurrent depression.

In this article, we consider the evidence from meta-analyses relating to MBIs in the mental health setting. The focus is on MBIs which have mindfulness training and transformation of mind as their main intention and activity, and therefore acceptance and commitment therapy (ACT) and dialectical behaviour therapy (DBT) have not been included. However, both of these approaches have elements of mindfulness within their multifaceted protocols, and the tight coupling to everyday goals, values and activities makes them a variant of mindfulness training that is well suited to working with clients who may find standardised MBIs difficult. As such, ACT work is included in the meta-analysis detailed below in relation to working with psychosis.

It is also beyond the scope of this article to consider the application of mindfulness-based approaches in the physical health setting.^15^ While there is plenty of evidence that MBSR (and some emerging protocols) can help alleviate a range of physical health conditions, it has been suggested the efficacy may be even greater in psychological disorders.^5,8^ We also make recommendations for best practice for those offering mindfulness training outside the current evidence. The overarching intention is to balance maintaining the integrity of standardised MBIs for certain specific populations with the very real explosion of interest in the area, and the multiple routes via which it is now accessed by clients in our clinical care. Careful formulation, based on a cognitive model, and clear intent are the guiding principles for this work.

## The evidence base

### Is MBCT effective?

Within the NHS, MBCT is recommended as a relapse prevention training for recurrent depression.^16^ Several controlled studies have shown that the addition of MBCT to treatment as usual (TAU) can reduce the chance of relapse by about 40-50% in participants who have experienced three or more episodes.^17-19^ The efficacy of MBCT appears at least comparable with maintenance antidepressant medication,^7,20^ making MBCT a viable alternative for those wishing to explore psychosocial ways to manage depressive illness. Early studies excluded individuals with a history of suicidal ideation, although recent work has explored the possibility that mindfulness training may also be helpful in these circumstances.^21,22^ For individuals with fewer than two episodes, or those whose depressive episodes are exogenously triggered (e.g. by stressful life events), the evidence is less compelling.^7,17^ An early onset of depressive illness and experience of childhood trauma or adversity appear to be factors that increase the efficacy of MBCT.^17,23^

From these studies, it is clear that the picture as it relates to clinical efficacy (specifically, preventing depressive relapse) is more complex than first thought and warrants caution in the indiscriminate application of mindfulness training as a ‘treatment’ for depression *per se*. Screening prior to group entry is a key part of the process of mindfulness training.^24^

### Is MBCT more effective than other approaches for depression?

One meta-analysis^5^ indicates the superiority of MBIs over relaxation, psychoeducation, supportive therapy and imagery-based approaches, but no additional benefit when compared with cognitive-behavioural therapy (CBT). The findings were broadly similar at up to 3 months' follow-up. In a well-controlled randomised controlled trial (RCT),^23^ where MBCT for depression was compared with a matched, active control condition (cognitive psychoeducation; CPE), there was no significant impact on the main outcome variable (relapse). However, for a sub-population of individuals in the study who had recurrent depression and childhood adversity, MBCT was superior in preventing relapse.^23^

Within the current literature, MBCT is indicated as an alternative offering to complement psychological (e.g. CBT) and pharmacological (antidepressant treatment) options, rather than necessarily being superior.^25^ It may be particularly useful for those with a more chronic presentation, whose early experiences perhaps did not include the modelling needed to develop healthy emotional awareness and management. Providing a choice for clients is also important, and particularly so for those who have received CBT and found it ineffective or those who do not easily fit into the CBT model. This might also include those whose mental health difficulties prevent an active, motivated engagement with a process aimed at challenging thought content (as in CBT), so-called ‘traditional psychiatric insight’.^2^ Working with thought processes rather than thought content makes it less necessary to have full insight. For example, in the work with psychosis, it may be possible to reduce the distress of auditory hallucinations by learning how to respond mindfully to a voice, while still maintaining a belief that the voice belongs to another entity.

### Is MBCT effective in other categories of mental illness?

Although large-scale, independently replicated RCTs are few and far between in other areas of mental health, one of the larger randomised studies comparing MBCT for bipolar disorder and TAU^26^ showed no significant difference between interventions with respect to relapse, mood symptoms or total number of episodes. However, there was a significant reduction in state anxiety in the MBCT group, also reported in Williams' work.^22^ Given that inter-episode anxiety is a crippling, often neglected feature of bipolar illness,^27,28^ ameliorating it could make a big difference in quality of life and social/occupational functioning. In this case, it is clear that MBCT is having an ‘effect’ *per se*, but the mechanism of action may be different in bipolar illness than in major (recurrent) depression. Two meta-analyses indicate that MBIs are helpful in the management of clinical anxiety^5,29^ and reductions in anxiety are also seen in non- or subclinical populations.^9,13^ A recent RCT showed no short-term advantage for MBCT over psychoeducation for generalised anxiety disorder (GAD).^30^

In the field of psychosis research, there has been a growing number of studies looking at MBIs, including the more broadly defined third-wave therapies that contain components of mindfulness such as ACT.^31,32^ Two meta-analyses have demonstrated that mindfulness training shows moderate short- and long-term benefits for positive symptoms and hospital admissions when compared with TAU,^32^ that effects are similar to other active interventions (such as CBT), and that there is lower attrition.^31^ This may be linked to the mindfulness stance which does not challenge the veracity of abnormal thinking and perceptions, but works with the process of how these emerge and are managed.^2^

A number of pilot feasibility studies report results from standardised (or modified) MBIs administered to different target populations, or MBIs with novel protocols.^1-3^ These approaches target specific features of a mental illness (e.g. auditory hallucinations or the impulse to binge eat) or postulate alternative methods such as mindful movement as an entry point to mindfulness.^33^

With these adaptations emerging, there is ongoing discussion in the field about more cognitively formulated mindfulness training (e.g. targeting specific mental processes such as rumination) *v*. more generic mindfulness training that teaches ‘techniques’.^34^ The latter may have a different type of clinical value – developing the ability to manage ‘stress’ or anxiety is likely to have a positive effect on almost any physical or mental health condition.

### Unexpected consequences

The range of outcome measures described in the cited studies points to the multiple routes through which mindfulness training may have its effects. In MBIs, symptom reduction is rarely the target. Instead, the training is about changing the relationship to symptoms and, specifically, becoming more curious (in a compassionate way) about mental habits.^35^ If the previous relationship to symptoms was one of avoidance, then it is quite likely that mindfulness will make the symptoms appear ‘worse’ as the individual is more aware of them, relating to them in an engaged but relaxed manner. However, it is likely that even those who experience ‘more’ symptoms will be less distressed by them following mindfulness training.

There is ongoing discussion in the field and the popular press about whether mindfulness training may be harmful for some people.^17,36^ There are concerns that individuals with certain difficulties (such as depersonalisation and dissociative disorders, trauma, psychosis and severe eating disorders) may struggle with standard MBI protocols. However, Chadwick's^2^ elegant work with people with psychosis indicates that with careful formulation, adaptations and a high degree of compassion and skill, it is possible for clinicians to work mindfully with these populations. In the case of delusional beliefs and auditory hallucinations, it is not necessary to change the belief about the voice in order to create a different relationship to it through mindfulness. Working directly with the body as a starting point may not be appropriate for certain of the eating disorders (e.g. anorexia) and trauma. However, adaptations (e.g. using movement) may be an entry point.

As this field progresses, it will be vital that those teaching and training others in mindfulness are themselves open, non-reactive and non-judgemental about instances where mindfulness ‘has not worked’. Part of best practice is sharing what went wrong in order for us to learn more about cases where mindfulness training may need further adaptation or may not be useful. Creating a culture of acceptance around unexpected outcomes would be a good starting point for teams or clinics considering any form of mindfulness training. Among professionals in the field, modelling the principle of gentle, kind, open curiosity to whatever happens (even if it is an ‘unexpected effect’ in a participant) is vital. The stability of this stance is directly related to the personal experience of the professional.

### The experience of the facilitator

The facilitator's own experience of mindfulness is a central factor to help work through (mindfully) any unusual or unexpected effects arising from mindfulness practice. Less experienced facilitators may be working on the edge of awareness of their own, more deeply embedded, habits or emotional reactivity and thus may not yet have the requisite steadfastness, non-reactivity and non-judgement when they meet this in their clients. The ability to hold a curious, open and accepting stance, even when things are not as we wish them to be, are qualities a mindfulness practitioner and teacher develops over time. This eventually leads to a position whereby it is possible to have a strong emotion or experience a strong (usually ‘negative’) mental habit without getting drawn into it or trying to make it be different or ‘go away’. This skill, developed through repeated practice, allows one to trust that it is possible to not react or judge, even when we see we are being unskilful in our actions or communications.

There may be moments when the client needs to see and trust that they can maintain the observer position in such instances. Conversely, there may be times when it is necessary to stop practising mindfulness. If there are dramatic changes in either internal (mental) or external (life) contexts that make the endeavour uncompassionate, forced or with a sense of too much expectation, this may be time to pause and reconsider intentions.

While personal experience of mindfulness is essential for those who wish to deliver MBIs in the clinical setting, the ability to teach mindfulness in a group setting requires further skills and training. How mindfulness is taught seems to be as important as what is taught.^34^ The impact of the facilitator's own mindfulness experience and what is referred to as the ‘embodiment of mindfulness’ in the teacher appears critical to the observed clinical/therapeutic effects.^5,37^ The MBI-Teacher Assessment of Competency (MBI-TAC)^37^ has been proposed as a way to maximise the effectiveness of MBIs, and to ensure the quality of teaching in these clinical settings. It considers six domains of competence to teach MBIs: (1) coverage, pacing and organisation of session curriculum; (2) relational skills; (3) embodiment of mindfulness; (4) guiding mindfulness practices; (5) enquiry; and (6) holding a group. Box 1 outlines the key recommendations from the UK Network for Mindfulness-Based Teacher Training Organisations and all healthcare workers considering using mindfulness as a therapeutic intervention are advised to be familiar with these.^24,37^

The reader is alerted to the therapeutic potential of therapist mindfulness alone, which is an essential starting point for those who wish to use mindfulness clinically but have yet to complete a formal teacher training. Studies indicate that mindfulness training for therapists enhances core therapeutic skills,^38-40^ and has a positive impact on patient experience^41^ and on clinical outcomes.^42^ All this before you have even ‘done’ any mindfulness to the client!

Therefore, standard MBIs appear effective for certain conditions and at certain times. A number of outstanding challenges remain, including the provision for wider implementation of MBCT in the NHS, the training of therapists to deliver MBCT, ongoing discussion about the measurement of mindfulness as a construct,^43^ and the exact mechanism of change.^35,44-46^ There are unexpected effects that have to be (mindfully) attended to, and the experience of the facilitator seems to be a critical component. However, alternative MBIs – targeting different populations and using different methods – appear promising, and a huge variety of resources are now widely available in the public domain. We will now explore a number of suggestions for those considering working outside the evidence base.

**Box 1** Recommendations from the UK Network for Mindfulness-Based Teacher Training Organisations

Mindfulness training should include:

- familiarity and personal experience with the mindfulness practice they will teach;
- minimum duration of 12 months.

Background requirements:

- professional qualification in health, education or social care (or equivalent life experience)
- knowledge and experience of the target populations.

If delivering mindfulness-based cognitive therapy (MBCT):

- knowledge of associated research, psychological processes and evidence-based practice
- appropriate clinical training (if delivering to a clinical population).

Ongoing requirements:

- personal daily practice and annual meditation retreats
- continuous development through contact with other practitioners/teachers and through supervision (which includes reflecting into personal experience and receiving feedback)
- commitment to further training and update
- adherence to appropriate ethical framework.

## Working outside the evidence base

### The real-world clinical context

Models of ‘stepped’ approaches, including ‘low-dose’ mindfulness, have been suggested.^47^ These modified forms of MBI, which might be considered more lifestyle oriented, informal, practice-based approaches,^47^ appear beneficial for working adults,^48,49^ healthcare workers^50^ and general practitioners.^51^ In one meta-analysis, mindfulness and acceptance-based self-help (including books and online courses, but not apps) reduced depression and anxiety with small to moderate effect sizes, across a range of clinical and non-clinical adult populations.^13^ Evaluation of mindfulness apps is ongoing, with promising early results,^52^ but still in the early stages.^53^

Given the ever-widening opportunities to access various types of mindfulness training, and the currently quite specific and limited access to MBCT in the NHS setting, might clients benefit from more generic mindfulness training skills? If so, what competencies are needed to help support this endeavour? The intention will be necessarily distinct from that of a closed, group MBCT for recurrent depression. But what are the possible benefits and risks? An increasingly wide range of mental health professionals (medical, allied health, third sector, peer supporters, experts by experience) are sharing mindfulness in a variety of ways and with varying levels of supervision.^54^ One of the main challenges is to understand who needs what training to deliver (what type) of mindfulness intervention, and with what intent. Box 2 shows some scenarios that may be familiar to the reader. While these may provide initial ‘touch points’ with mindfulness, they are also instances where a lack of clear intention and poor expectation management may lead to disappointment and/or confusion.

It is necessary to manage the tension between allowing clients access to methods of psychological development that may be helpful (and are readily available to the general public), and upholding the professional ethics of ‘do no harm’ and operating within professional competencies.^55^ Having, and communicating, a clear and specific intention is vital. Related to this, clarity around the language and a deeper exploration of what is meant by ‘mindfulness training’ may be helpful. If you are offering an informal-based mindfulness training (drop-in sessions, apps, books etc.) the intentions, level of practice and depth of work is likely to be different than during a closed, 8-week training programme with regular 45 min practice sessions. Working systemically with mindfulness will necessarily have a different type of impact than working with the individual.

**Box 2** Clinical scenarios of mindfulness outside the standardised mindfulness-based interventions (MBIs)

- Clients asking clinicians to recommend apps, online training and/or self-help books
- Clients and carers asking whether a mindfulness course is suitable for their situation (if not depression in remission)
- Offering or suggesting to clients a mindfulness drop-in, peer-led or introductory session
- Offering bespoke mindfulness for groups where there is sparse evidence and less theoretical rationale
- Considering adapted mindfulness training courses (e.g. shorter-duration, low-dose variants of standardised MBIs)
- Enthusiastic mental health workers, healthcare assistants etc. who wish to start up a mindfulness group on a ward or in a service
- Adjunctive use of mindfulness tools in the context of another type of therapy
- Augmenting what is currently offered in psychoeducation courses by introducing mindfulness exercises
- Incorporating mindfulness into everyday staff activities (e.g. explicit reference to mindful principles as part of a referral meeting process or team debriefs)

### A proposed framework

**Fig. 1:**
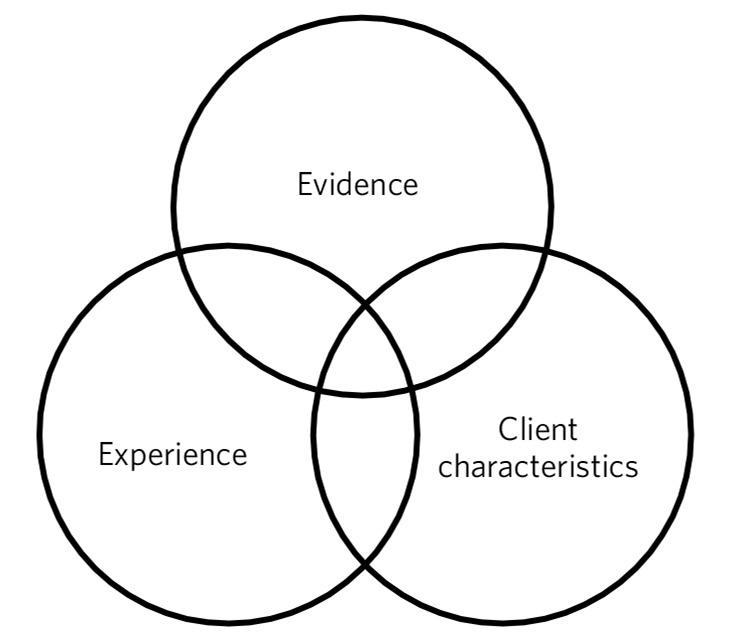
Three areas to consider if you are working with mindfulness outside the standard protocols: (a) the evidence base and intention of the work; (b) the characteristics of the client group and context; and (c) the facilitator's experience.

This article proposes a three-part framework (Fig. 1) to guide thinking in this emerging area. The overarching intention is to help those delivering new variants of mindfulness training in the mental health setting to do so as mindfully as possible. On this basis some preliminary recommendations are made below. Clinicians might consider rating the evidence, experience or client characteristics on a scale of 0-10. This will help guide decisions and also indicate where it is necessary for more evidence, supervision/training or thought to be given to a decision to implement mindfulness in each case.

## Recommendations

### Recommendation 1: understand the evidence (or lack thereof)

#### Familiarisation with the evidence base

Be aware of the evidence and stay up to date as the field is rapidly expanding – for example, sign up for the *Mindfulness Research Monthly* from the American Mindfulness Research Association (https://goamra.org/publications/mindfulness-research-monthly/). While assessing the literature, pay close attention to adaptations for particular groups, and understand the cognitive/emotional rationale for the adaptation. Look at the specifics: type and duration of exercises, number and duration of sessions; standard, adapted or novel protocol; the physical/psychological context of the intervention, level of support available; and teacher competencies. Take note of any unusual or unexpected effects reported by the authors, or other areas highlighted as warranting caution or specific expertise.

Check the intention of the interventions as you review them. Was mindfulness offered as a targeted, formulated intervention to work with specific symptoms or as a more general skills training (e.g. to manage stress/anxiety)? How similar or different is this to your context and proposed work? At what stage of illness was the intervention offered? MBCT is offered during a period of remission from depression. This provides a chance to develop skills during a period of (relative) mental calm, so they are ready to be implemented when mental states start to change. If clients are acutely unwell, there might be a case for working systemically (see Singh's work with staff^56^ and carers^57^).

#### Talk to experts by experience

If possible, talk to those in your client group who have experience of using mindfulness (e.g. recovery college workers, peer supporters, peers working in third-sector organisations). This ‘anecdotal’ evidence can offer unique insights or observations from individuals who have made their own adaptations. Talk to other facilitators or teachers who have either clinical or mindfulness expertise with a particular client group to get their insights. Given the importance of personal practice and of depth of understanding of the practice of mindfulness and its application to the specific mental struggles associated with different ‘psychopathologies’, it may be that a so-called ‘peer’ expert, with both personal experience of mental ill health and training in mindfulness, is the most suitable person to learn mindfulness from. In our experience, clients frequently ask facilitators ‘Have you used mindfulness to work with x or y mental states'? If someone with direct, personal experience of using mindfulness – for example, to work with voices – can support someone in a similar situation, this may be an even more powerful way to work. How to best support and train these peer teachers is an ongoing discussion in the field. Clearly their personality experience is a strength (but also a vulnerability). Teacher guidelines for ‘professionals’ as described in Box 1 are extremely valuable; however, it may be helpful in future to develop guidelines for peer teachers of mindfulness (this work is ongoing at the Mindfulness Centre of Excellence, London, and more information is available from the authors on request).

#### Evaluate your intervention

Gather your own qualitative (e.g. feedback form) or quantitative (e.g. pre-/post-ratings) evidence using measures ideally co-designed with your client group. This might include self-rated ‘stress bubbles’ (as used in Russell^3^), measures of mindfulness, or a more functional analysis, recording whether practical tasks (such as getting out or on a bus) were made easier or less distressing as a result of mindfulness training. Allow space to mindfully evaluate any unusual or unexpected effects of the intervention. Consider ways you can share your evaluation or observation so others can benefit from your experience (blogs, newsletters, short reports, conference abstract or paper).

### Recommendation 2: client characteristics

Mindfulness requires the ability to become aware of mental and physical sensations, as they unfold moment by moment. Necessarily, this requires: (a) some form of paying attention; (b) an ability to engage with, yet not react to, physical (including emotional) and mental experiences; and (c) a mind oriented towards non-judging, acceptance and nurturing. Clients in the clinical setting will have a range of capacities in these attentional, emotion regulation and attachment domains. Low scores in all three domains may indicate something other than mindfulness as more appropriate. Some screening questions provided by Dobkin *et al*^36^ indicate groups where caution and adequate preparation for the group would be helpful.

Adaptations to take into account reduced attentional capacities can be found in the work exploring mindfulness with people who have attention-deficit hyperactivity disorder (ADHD),^58^ whereas Chadwick^2^ describes modifications to support those experiencing the symptoms of psychosis. Use of external objects or mindful movements^3^ might initially help individuals who struggle to sit still and look inwards. Mindfulness skills for distress tolerance, as outlined in the DBT model,^59^ may be more appropriate for those experiencing intense affect.

#### Managing expectations

Managing expectations is essential and will be informed by your knowledge of the evidence base and how this relates to what you are offering. The different intent behind targeted *v*. generic training is ideally shared, so there are realistic expectations about what is involved (in terms of practice) and likely outcomes. Myth-busting around mindfulness as ‘relaxation’ and the ‘double-edged sword’ of awareness (e.g. increased ‘symptoms’) will further allow clients to enter into mindfulness training with a better understanding of what is possible and what is being asked.

#### Management of unusual experiences

Consider, based on the reported literature, your own experience, your knowledge of the client group, and any possible unusual or unexpected effects that might arise. Make a plan for how these might be recorded and managed mindfully. Consider at what point you would suggest that someone stop practising mindfulness.

### Recommendation 3: facilitator experience

An emerging theme from the literature is that the embodiment of mindfulness in the person facilitating the training is vital. We are each at our own unique place in the development of our personal mindfulness skills, but if we wish to teach or share mindfulness with others, there needs to be an active commitment to self-reflection and ongoing professional development. An initial reflection might be around your own interest in, and path to, mindfulness. Was it through yoga practice, a secular or a Buddhist group? How does this training affect what you offer in the clinical setting? What might need modification or dropping altogether? As a general rule, teach what you have personal experience of, and do not ask anyone to do a practice you have not done yourself, under a variety of conditions (ranging from calm to under duress). What barriers do you come across? How can this learning be appropriately shared or modelled in your work?

#### Intentions and expectations

Be clear in your own mind as to what type/level of mindfulness you are confident to deliver. If you are working outside the evidence you are likely delivering a more generic, technique-oriented mindfulness training to reduce stress and reactivity. Have a good knowledge of what you are *not* doing, and practise clear communication about your intentions.

#### Facilitator skills audit

A mindful reflection on your own competence as someone teaching or sharing mindfulness requires an ongoing compassionate acceptance of your own limitations and growth areas. It requires the courage to continue to develop personally as well as developing as a facilitator/teacher of mindfulness. With this spirit in mind, one suggestion is to use the competencies outlined in the MBI-TAC^9^ as a framework against which to realistically (but gently) assess your current capabilities. Where possible, ask an external observer (e.g. a co-facilitator) or your participants to give you feedback. Consider the most pressing continuing professional development (CPD) need for this work and create your own package. This could include formal training, attending a course taught by another teacher, retreats, exploring online learning, or listening to different teachers via online resources. Ideally, ask for guidance from a more senior teacher in your area if CPD is not available. Be clear with your intention as you train – is this development of your personal mindfulness or development for teaching mindfulness? – and look out for the ‘mindfulness teacher’ mental habit when doing your personal work. This is the often reported phenomenon where mindfulness teachers, during their own training, get lost in thought about how they themselves will teach or explain the concept they are currently learning about. Thus they have left experiential learning and are in the conceptual mind space.

#### Support

Consider what support or supervision is available to you, for both your personal development and your teacher development. Are there colleagues, mentors or more senior practitioners in your network with whom you can discuss any challenges that arise? If not, how and where can you access this (e.g. mindfulness practitioner/teacher networks, physical or online meetings)? A protocol for mindfulness self-reflection, which can be done at the group or individual level, is provided by Russell & Tatton-Ramos.^60^

## Summary and conclusions

When used as a clinical intervention for major depressive disorder, there is good evidence that MBCT can prevent relapse to a degree that is at least similar to currently available treatments. It may have advantages for particular subgroups of depressed individuals with more long-standing, recurrent depressive illness and childhood adversity. Evidence for efficacy in other domains of mental ill health is less convincing, but it is emerging. Although strong evidence exists for the application of mindfulness in the management of anxiety (generalised), this work does not seem as prevalent in the UK setting. This may be because CBT approaches are very effective for anxiety disorders so there is less of a driver to find alternatives.

The breadth of ‘mindfulness interventions’ continues to grow, from standardised protocols to peer-led drop-ins, apps and self-help materials. Navigating this growing landscape in a way that is true to the transformational possibilities of mindfulness and that allows clients to connect to mindfulness in a meaningful and healthy way presents some challenges. Some recommendations have been made here to help in this endeavour. Specifically, to know the state of the evidence, to be aware of relevant client characteristics, and to know your own limitations as a teacher or facilitator of mindfulness. Continuing personal and professional development is essential and will have an impact on efficacy. These are exciting times as the impact of mindfulness training spreads throughout our health services, offering a chance for both staff and clients to benefit and improve their mental ‘wealth’. However, it is most important that this endeavour is conducted in a mindful way – paying attention, on purpose, moment by moment and without judgement.
